# Ectopic Breast Tissue of the Vulva in a Postmenopausal Woman

**DOI:** 10.1155/2017/7581750

**Published:** 2017-10-12

**Authors:** Sasha Mikhael, Wesley Nilsson, Kruti Patel, Sherri Graf

**Affiliations:** ^1^Providence-Providence Park Hospital, Michigan State University College of Human Medicine, USA; ^2^American University of the Caribbean, USA

## Abstract

Ectopic breast tissue is a rare condition caused by remnants of the mammary ridges that fail to involute during embryologic development. To date, only 39 cases have been reported in the literature worldwide. Here, we report the 40th case of a 69-year-old G3P2 postmenopausal Caucasian woman who presented with complaint of vulvar swelling. Biopsy of the mass revealed the presence of a benign mammary gland-like adenoma which was completely excised shortly thereafter. Clinical presentation of vulvar breast tissue is highly variable depending on the amount of breast tissue developed and its functionality. Diagnosis is ultimately made by tissue biopsy and histopathologic examination. Due to the scarce evidence that exists pertaining to supernumerary breast tissue located on the vulva, specific management guidelines are lacking. Since this ectopic tissue serves no function, but rather may provide a source for future malignancy, surgical excision is recommended.

## 1. Introduction

During embryologic development of the breast at approximately four-week gestation, paired ectodermal thickenings produce the mammary ridges on the ventral surface of the embryo, which extends from the axillae to the medial thigh. Incomplete resorption of these milk lines can lead to ectopic remnants of breast tissue. The presence of ectopic mammary tissue is rare with a prevalence of 1-2% in the general population and primarily found in the axillae. The occurrence of accessory breast tissue located in the vulva is extremely rare with only 39 cases reported to date, worldwide [1], only 10 of which are unilateral. Clinically, vulvar breast tissue may present with vulvar swelling or as a vulvar “mass.” Ultimately, to achieve a definitive diagnosis, a biopsy with histopathologic confirmation is required. Here we describe a case of unilateral ectopic mammary tissue of the vulva. To the best of our knowledge, this is the 40th reported case involving supernumerary breast tissue in the vulva and only the 11th to be unilateral.

## 2. Case Report

**Figure 1:**
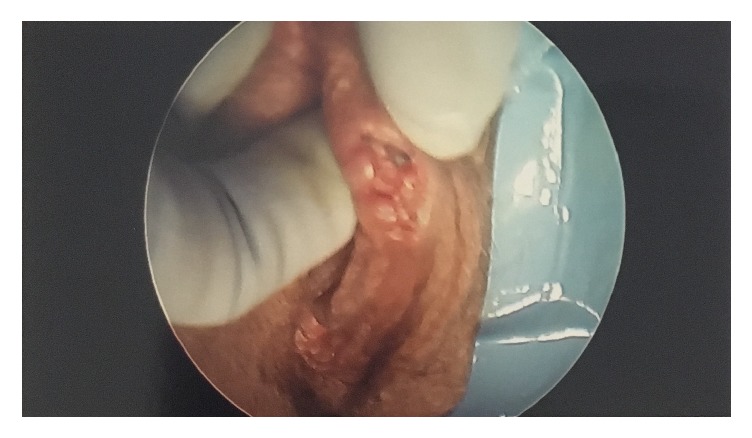
Previously sampled right vulvar mass containing mammary gland-like tissue taken by hysteroscopic camera. Image taken with permission from patient at time of surgery.

**Figure 2:**
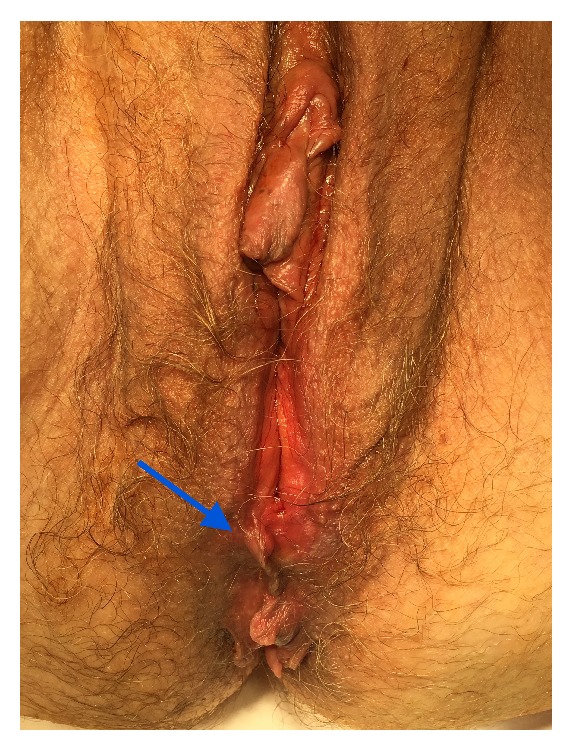
Ectopic breast tissue noted on the vulva as pointed out by the arrow. Image taken with permission from patient at time of surgery.

A 69-year-old G3P2 Caucasian female presented with complaints of vaginal dryness and a bump on her vulva that was bothersome, though it did not cause any pain or discomfort. On physical exam, a 2 cm irregular nodular mass was noted on the right vulva at the right minora juncture, which was biopsied (Figure 1). The pathology report concluded the presence of nodular proliferation of mammary gland-like tissue with florid hyperplasia, cystically dilated ducts, and apocrine metaplasia involving even the deep resection margins. Due to an incidentally found thickened endometrium and fibroid uterus previously noted on ultrasound, the patient was scheduled for complete excision of the ectopic breast tissue during her planned procedure for a hysteroscopy and curettage. Following vulvar biopsy of mammary tissue (Figure 2), the patient underwent a deeper excision of the tissue measuring 16 mm at the vaginal fourchette. Histopathology of endometrial tissue samples indicated polypoid fragments of squamous mucosa with acute chronic inflammation suggestive of Asherman syndrome and that of the vulvar excision demonstrated focal benign ducts, confirming the presence of ectopic mammary tissue remnants with clear margins. The patient was seen postoperatively and the excision site had notable granulation tissue indicative of adequate healing.

## 3. Discussion

Ectopic breast tissue of the vulva is an extremely rare condition that was first described by Hartrung in 1872 with only a handful of cases reported since that time, including benign and malignant cases. Development of supernumerary breast tissue exists due to failure of the milk lines to involute, which run the length of the ventral body wall from the axillae to the groin in a curvilinear pattern [2]. Based on previously reported cases, it appears that presentation is highly variable, based on the level of developed breast tissue observed and its functionality [3]. Nipple and areola may be apparent; however, in other cases, and more commonly, when ectopic breast tissue in the vulva is primarily composed of adipose, it can be mistaken for a lipoma [1, 4].

Vulvar breast tissue is also subject to physiologic and pathologic changes. Though this condition is congenital, it does not become clinically apparent until enlargement occurs, which results from hormonal influences [2]. Therefore most cases are noted during pregnancy or lactation. In some cases, the glands remain dormant throughout puberty and pregnancy, as noted in our patient where ectopic mammary tissue was not discovered until long after menopause. Though initially believed to be a lipoma, a misdiagnosis commonly made, histopathology of the tissue confirmed the presence of mammary glands.

Unfortunately scarce evidence exists in the literature regarding the presence of vulvar breast tissue due to its rare incidence. As a result, little guidance exists on the management approach for these patients. Vulvar breast tissue ultimately serves no function but rather creates potential for disease since, like normal breast tissue, it may undergo neoplastic changes. Consequently, even in benign cases, once discovered, it should be surgically excised immediately [1, 2]. No guidelines exist regarding how to approach follow-up in these patients in terms of whether to screen for ectopic breast malignancy or recurrence of ectopic tissue; however the general consensus is to ensure that all margins of excised tissue are clear of mammary glands.
